# Risk assessment and clinical prediction model of planned transfer to the ICU after hip arthroplasty in elderly individuals

**DOI:** 10.1186/s12893-023-02204-2

**Published:** 2023-10-07

**Authors:** Jianguang Sun, Lue Huang, Yali Yang, Hongxing Liao

**Affiliations:** Meizhou People’s Hospital, Meizhou, China

**Keywords:** Hip arthroplasty in elderly individuals, Planned transfer to the ICU, Logistic regression analysis, Clinical prediction model

## Abstract

**Background:**

With the development of hip arthroplasty technology and rapid rehabilitation theory, the number of hip arthroplasties in elderly individuals is gradually increasing, and their satisfaction with surgery is also gradually improving. However, for elderly individuals, many basic diseases, poor nutritional status, the probability of surgery, anaesthesia and postoperative complications cannot be ignored. How to reduce the incidence of postoperative complications, optimize medical examination for elderly patients, and reasonably allocate medical resources. This study focuses on the construction of a clinical prediction model for planned transfer to the ICU after hip arthroplasty in elderly individuals.

**Methods:**

We retrospectively analysed 325 elderly patients who underwent hip arthroplasty. The general data and preoperative laboratory test results of the patients were collected. Univariate and multivariate logistic regression analyses were performed to screen independent influencing factors. The backwards LR method was used to establish the prediction model. Then, we assessed and verified the degree of discrimination, calibration and clinical usefulness of the model. Finally, the prediction model was rendered in the form of a nomogram.

**Results:**

Age, blood glucose, direct bilirubin, glutamic-pyruvic transaminase, serum albumin, prothrombin time and haemoglobin were independent influencing factors of planned transfer to the ICU after hip arthroplasty. The area under the curve (AUC) of discrimination and the 500 bootstrap internal validation AUC of this prediction model was 0.793. The calibration curve fluctuated around the ideal curve and had no obvious deviation from the ideal curve. When the prediction probability was 12%-80%, the clinical decision curve was above two extreme lines. The discrimination, calibration and clinical applicability of this prediction model were good. The clinical prediction model was compared with the seven factors in the model for discrimination and clinical use. The discrimination and clinical practicability of this prediction model were superior to those of the internal factors.

**Conclusion:**

The prediction model has good clinical prediction ability and clinical practicability. The model is presented in the form of a linear graph, which provides an effective reference for the individual risk assessment of patients.

## Introduction

With the development of society, the number of elderly individuals undergoing hip disease surgeries is gradually increasing, and surgical technology is also constantly improving. Elderly patients have systemic dysfunction, decreased tolerance to surgery and decreased ability to regulate stress, often combined with basic diseases such as ischaemic heart disease, COPD, dementia, and diabetes, which increase postoperative complications and mortality [1]. It is estimated that one in every 30 surgical patients requires intensive care after total joint replacement [2]. The intensive care unit (ICU) is particularly important for the life security of high-risk patients [3].

An increasing number of clinicians are paying attention to how to reduce surgical complications, improve the medical service experience of patients and their families, shorten hospitalization time and reduce hospitalization costs. Previous studies have mostly discussed the related factors influencing unplanned transfer to the ICU after hip arthroplasty, but there is still a lack of valuable clinical consensus on planned transfer to the ICU after surgery [4]. We found in clinical work that unplanned transfer to the intensive care unit will cause problems such as insufficient ICU beds, lack of medical staff, and improper doctor-patient communication. This study focuses on the related risk factors for postoperative planned transfer to the ICU and constructs a prediction model to improve the advantages of perioperative risk assessment, reduce postoperative complications, reduce hospitalization time, and improve the medical service experience of patients and their families.

## Methods

### Research subjects

**Fig. 1:**
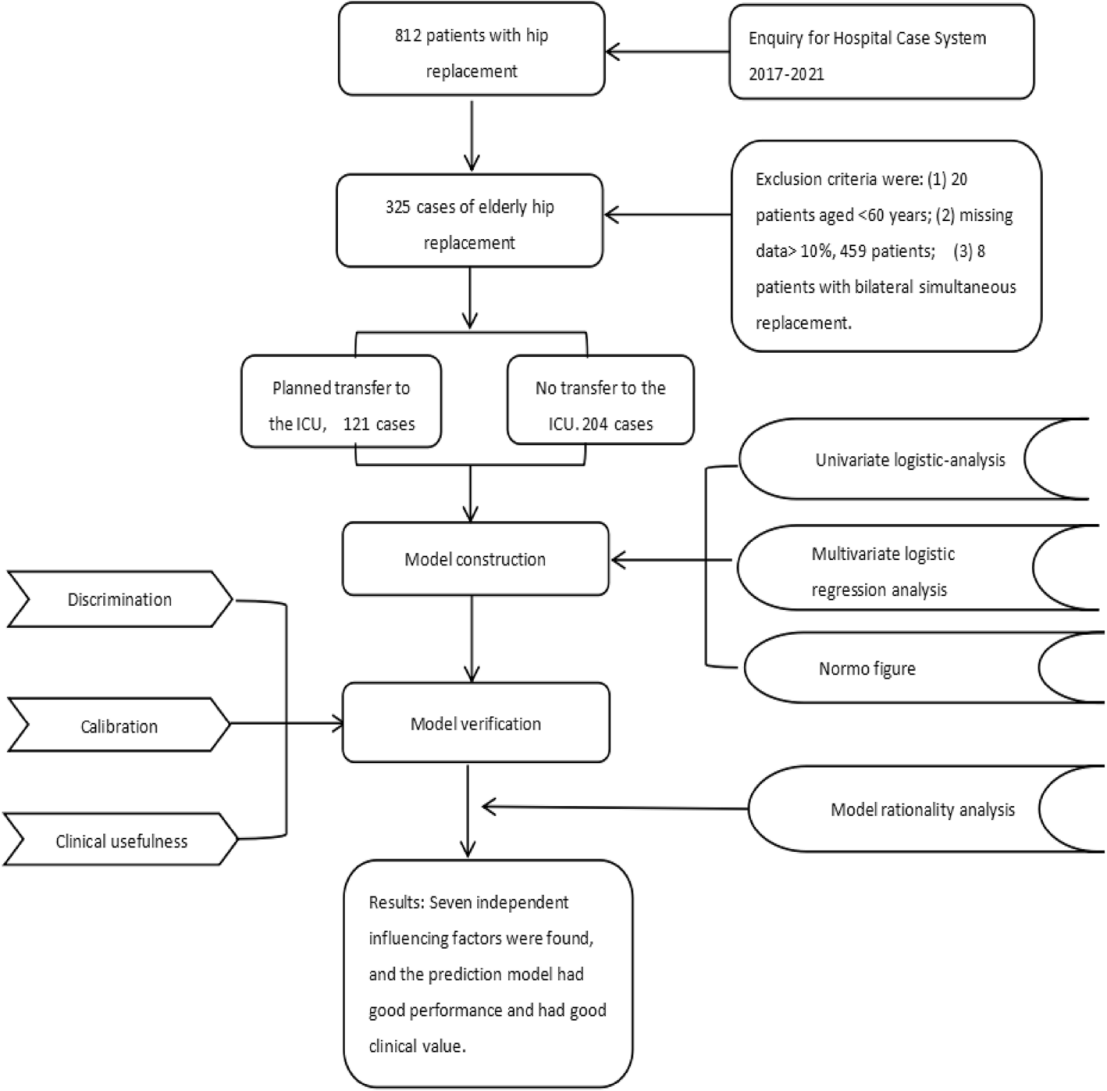
Flow chart of patient screening

We extracted data from 812 elderly patients with hip arthroplasty from the hospital medical record database from 2017 to 2021 and included 325 patients in the data analysis, including 121 patients who were planned to be transferred to the ICU and 204 patients who were not transferred to the ICU. All hip replacement procedures were performed by the same physician with a senior professional title. The inclusion criteria were as follows: (1) patients aged ≥ 60 years with hip arthroplasty; (2) available patient preoperative test results, anaesthesia and other data; (3) unilateral surgery; and (4) patients and their families agreed to provide clinical data as the research content. The exclusion criteria were as follows: (1) patients < 60 years old; (2) simultaneous bilateral hip replacement; and (3) missing data > 10%. The method of filling in the mean value for some missing important data. The flowchart is shown in Fig. 1.

This study was supported by the Ethics Committee of Meizhou People’s Hospital, and the procedures performed in this study are consistent with the 1964 Helsinki Declaration.

#### Research indicators

Research indicators are usually based on previous studies, statistically significant indicators or clinically relevant indicators. This study included a total of 25 indicators: age (years), gender (male, female), preoperative pulse (times/min), preoperative anaesthesia plan (general anaesthesia or spinal epidural anaesthesia), blood pressure (mmHg), comorbidity index (CCI), red blood cells (10^9^/L), haemoglobin (g/L), haematocrit (%), platelets (10^9^/L), prothrombin time (seconds), international standardized ratio, thrombin time (seconds), partial thromboplastin time (seconds), fibrinogen (g/L), K^+^(mmol/L), total protein (g/L), albumin (g/L), glutamic oxalacetic transaminase (U/L), glutamic-pyruvic transaminase (U/L), total bilirubin (µmol/L), direct bilirubin (µmol/L), urea nitrogen (mmol/L), and blood glucose (mmol/L).

#### Statistical methods

SPSS 25.0 software (SPSS Inc. Chicago, IL) and R 4.2.1 language were used to analyse the clinical data. The general data and baseline analysis of the patients were analysed by SPSS 25.0 software. The measurement data with a normal distribution are expressed as means ± standard deviations (SD), and comparisons between the two groups were performed using the independent sample t test. The measurement data with a skewed distribution are expressed as medians and interquartile ranges (IQR), and the Mann–Whitney U test was used to determine differences between groups. The counting data are expressed as frequencies (percentages), and the chi-square test was used for the analysis of counting data (Table 1). Univariate analysis and multifactor logistic regression analysis were used to construct a clinical prediction model. The final model was screened by the backwards LR method. Finally, ROC curves, calibration curves, and decision curve analysis (DCA) were used to assess the nomogram’s prediction accuracy. *P* < 0.05 was considered statistically significant.

**Table 1:** Comparison of general data between two groups of patients

	No transfer to the ICU (204)	Planned transfer to ICU (121)	*P*
CCI	0.659 ± 1.26	1.027 ± 1.344	0.113
Age	74.33 ± 8.64	80.68 ± 9.35	< 0.001
Gender(%)			
Female	125 (61.2%)	66 (54.5%)	1
Man	79 (38.7%)	55 (45.5%)	
GLU	6.25 ± 3.11	6.96 ± 2.85	< 0.001
SCR	97.90 ± 102.74	122.10 ± 160.10	0.451
BUN	7.17 ± 5.00	8.48 ± 5.51	0.034
DBIL	3.89 ± 2.34	5.77 ± 3.89	< 0.001
TBIL	13.46 ± 6.99	5.78 ± 3.89	0.001
ALT	21.91 ± 10.73	28.36 ± 22.27	< 0.001
AST	17.99 ± 10.70	22.36 ± 16.21	0.101
ALB	37.85 ± 4.68	33.91 ± 5.60	< 0.001
TP	66.37 ± 6.29	62.95 ± 7.15	< 0.001
K^+^	3.97 ± 0.55	3.94 ± 0.56	0.497
FIB	4.49 ± 1.39	4.91 ± 1.48	0.001
APTT	34.30 ± 5.80	34.13 ± 6.21	0.523
TT	12.67 ± 2.11	15.51 ± 2.60	0.383
INR	1.52 ± 6.65	1.13 ± 0.29	< 0.001
PT	12.61 ± 1.33	13.32 ± 3.15	< 0.001
PLT	239.61 ± 82.33	227.81 ± 98.67	0.077
HCT	37.39 ± 5.52	33.32 ± 6.34	< 0.001
RED	4.25 ± 0.79	3.75 ± 0.823	< 0.001
Pulse	82.47 ± 10.09	85.74 ± 12.71	0.145
HP	123.54 ± 19.47	110 ± 21.42	< 0.001
Anesthesia(%)			
General anesthesia	119 (58.3%)	64 (52.9%)	0.01
Spinal epidural anesthesia	85 (41.7%)	57 (47.1%)	
Blood pressure			
Systolic pressure	146.8 ± 85.83	139.35 ± 17.75	0.592
Diastolic pressure	83.0 ± 9.98	82.1 ± 11.89	0.317

*ICU* Intensive care unit, *CCI* Charlson comorbidity index, *GLU* Blood glucose, *SCR* Serum creatinine, *BUN* Usea nitrogen, *DBIL* Direct bilirubin, *TBIL* Total bilirubin, *ALT* Glutamic-pyruvic transaminase, *AST* Glutamic-oxalacetic transaminase, *ALB* Albumin, *TP* Total protein, *FIB* Fibrinogen, *APTT* Activated partial thromboplastin timepartial thromboplastin time, *TT* Thrombin time, *INR* International normalized ratio, *PT* Prothrombin time, *PLT* Platelet, *HCT* Hematocrit, *RED* Red blood cell, *HP* Hemoglobin

## Results

### Risk factors

R 4.2.1 language was used to perform logistic univariate analysis on 25 related factors, such as general data and preoperative laboratory test results of patients. The results showed that 15 of the 25 related factors were statistically significant, namely, age, urea nitrogen, pulse, haemoglobin, red blood cells, haematocrit, fibrinogen, total protein, albumin, prothrombin time, alanine aminotransferase, aspartate aminotransferase, total bilirubin, direct bilirubin, and blood glucose (Table 2). These 15 statistically significant indicators were included in the multivariate logistic regression. The results showed that age, blood glucose, direct bilirubin, alanine aminotransferase, serum albumin, prothrombin time, and haemoglobin were independent influencing factors for planned ICU transfer after hip arthroplasty in elderly patients (Table 3). We evaluated the multiple collinearity of the variable with the variance inflation factor (VIF).The general guideline is that VIF values above 5 or 10 suggest high multicollinearity and may require further investigation. We obtain that all VIF values are less than 2. It shows that there is no collinearity problem among the variables.

**Table 2:** Univariate logistic analysis of planned ICU transfer after elderly hip arthroplasty

Variable	B	SE	OR	CI	Z	*P*
CCI	0.097	0.076	1.10	0.95–1.28	1.283	0.200
Age	0.077	0.014	1.08	1.05–1.11	5.639	< 0.001
Gender	-0.277	0.232	0.76	0.48–1.2	-1.19	0.234
GLU	0.081	0.041	1.08	1–1.18	1.981	0.048
SCR	0.001	0.001	1.00	1–1	1.622	0.105
BUN	0.052	0.024	1.05	1.01–1.1	2.151	0.031
DBIL	0.206	0.045	1.23	1.13–1.34	4.539	< 0.001
TBIL	0.048	0.016	1.05	1.02–1.08	3.091	0.002
ALT	0.028	0.01	1.03	1.01–1.05	2.695	0.007
AST	0.032	0.01	1.03	1.01–1.05	3.125	0.002
ALB	-0.154	0.026	0.86	0.81–0.9	-5.916	< 0.001
TP	-0.075	0.018	0.93	0.9–0.96	-4.112	0.016
K^+^	-0.072	0.209	0.93	0.62–1.4	-0.344	0.731
FIB	0.213	0.081	1.24	1.06–1.45	2.635	0.008
APTT	-0.006	0.019	0.99	0.96–1.03	-0.291	0.771
TT	-0.034	0.051	0.97	0.87–1.07	-0.676	0.499
INR	-0.023	0.042	0.98	0.9–1.06	-0.532	0.595
PT	0.178	0.074	1.19	1.03–1.38	2.399	< 0.001
PLT	-0.001	0.001	1.00	1–1	-1.083	0.279
HCT	-0.106	0.021	0.90	0.86–0.94	-5.062	< 0.001
RED	-0.786	0.163	0.46	0.33–0.63	-4.818	< 0.001
Pulse	0.026	0.011	1.03	1–1.05	2.461	0.014
HP	-0.031	0.006	0.97	0.96–0.98	-5.059	< 0.001
Anesthesia	0.221	0.231	1.25	0.79–1.96	0.955	0.339
Blood.pressure	-0.017	0.229	0.98	0.63–1.54	-0.072	0.943

*ICU* Intensive care unit, *CCI* Charlson comorbidity index, *GLU* Blood glucose, *SCR* Serum creatinine, *BUN* Usea nitrogen, *DBIL* Direct bilirubin, *TBIL* Total bilirubin, *ALT* Glutamic-pyruvic transaminase, *AST* Glutamic-oxalacetic transaminase, *ALB* Albumin, *TP* Total protein, *FIB* Fibrinogen, *APTT* Activated partial thromboplastin timepartial thromboplastin time, *TT* Thrombin time, *INR* International normalized ratio, *PT* Prothrombin time, *PLT* Platelet, *HCT* Hematocrit, *RED* Red blood cell, *HP* Hemoglobin

**Table 3:** Multivariate logistic regression of planned transfer to ICU after elderly hip arthroplasty

Variable	B	SE	OR	CI	Z	*P*
Age	0.061	0.016	1.06	1.031–1.098	3.827	< 0.001
GLU	0.063	0.044	1.07	0.982–1.167	1.450	0.147
DBIL	0.114	0.048	1.12	1.023–1.236	2.367	0.018
ALT	0.033	0.012	1.03	1.011–1.061	2.675	0.007
ALB	-0.075	0.029	0.93	0.874–0.982	-2.541	0.011
PT	0.115	0.063	1.12	0.995–1.287	1.818	0.069
HP	-0.015	0.007	0.98	0.97–0.999	-2.056	0.040

*GLU* Blood glucose, *DBIL* Direct bilirubin, *ALT* Glutamic-pyruvic transaminase, *ALB* Albumin, *PT* Prothrombin time, *HP* Hemoglobin

#### Prediction model construction

**Fig. 2:**
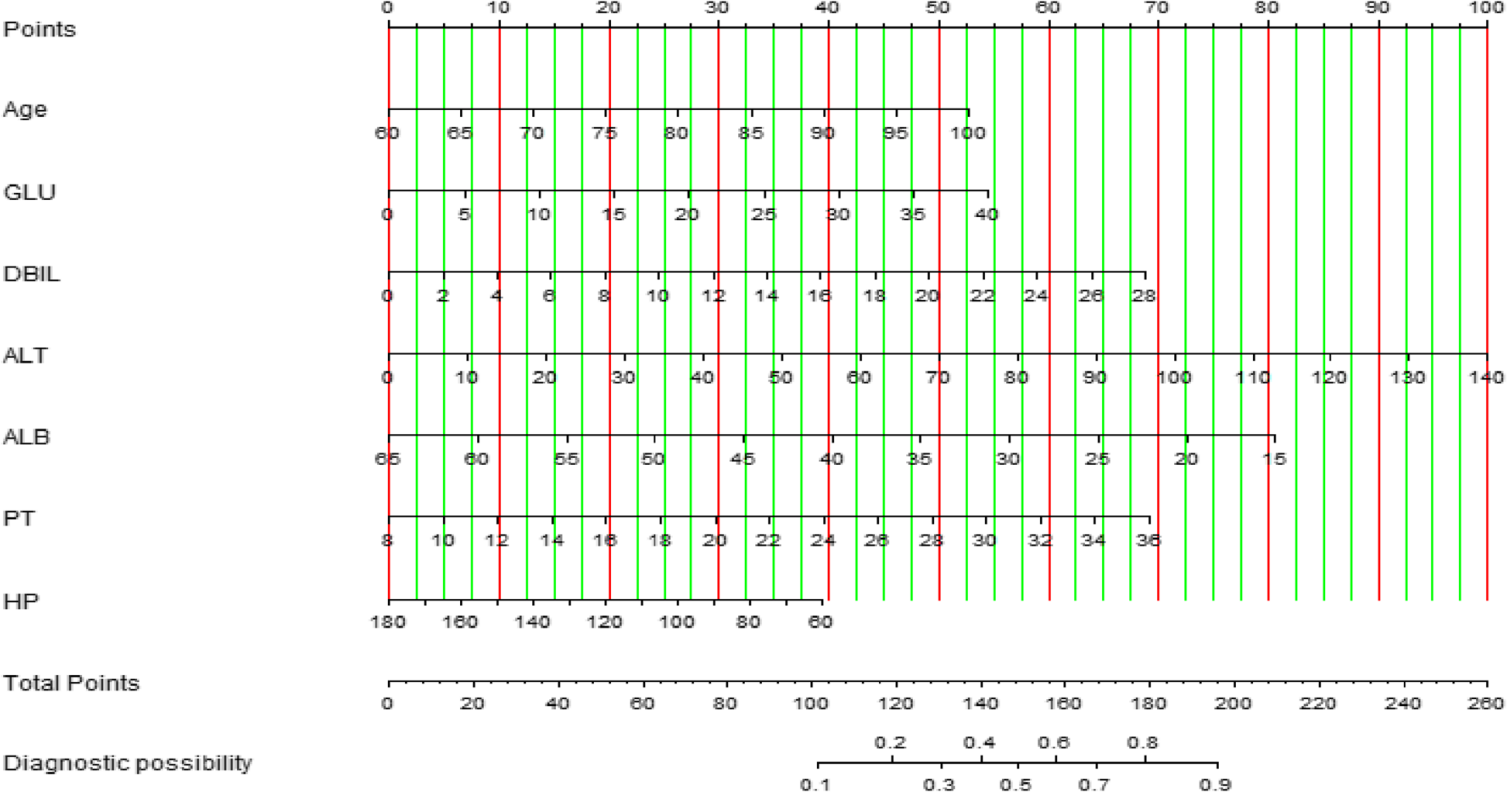
Nomogram of the prediction model. GLU Blood glucose, DBIL Direct bilirubin, ALT Glutamic-pyruvic transaminase, ALB Albumin, TP Total protein, PT Prothrombin time, HP Hemoglobin

The risk factors in multivariate logistic regression were analysed by R language and screened by the backwards LR method to establish a visual prediction model, namely, a nomogram. A nomogram is able to personalize a prediction so that it can identify and assess the risk of each patient [5] (Fig. 2). A nomogram converts the regression equation into a visual graph that is easy to understand, which makes the results of the prediction model more readable and facilitates patient risk assessment. In Fig. 2, the indicator on the left represents the independent variable, with each variable representing the axis corresponding to the line chart and indicating the score for the variable. The total score of each variable plus the total score of the corresponding total score table corresponds to the diagnostic possibility of planned transfer to the ICU after hip arthroplasty.

## Evaluation and verification of the model

### Discrimination

**Fig. 3:**
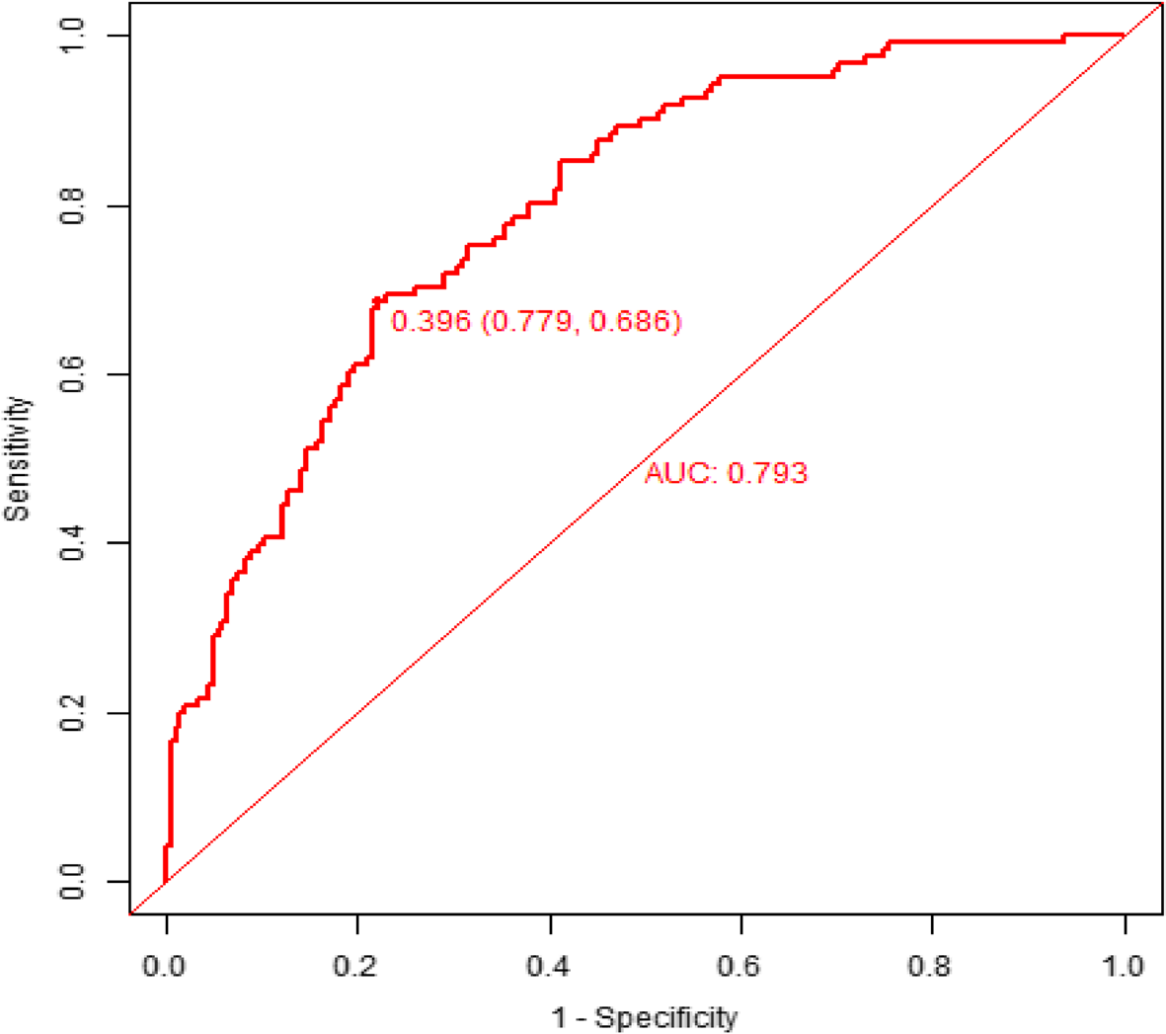
The ROC curves of the prediction model

**Fig. 4:**
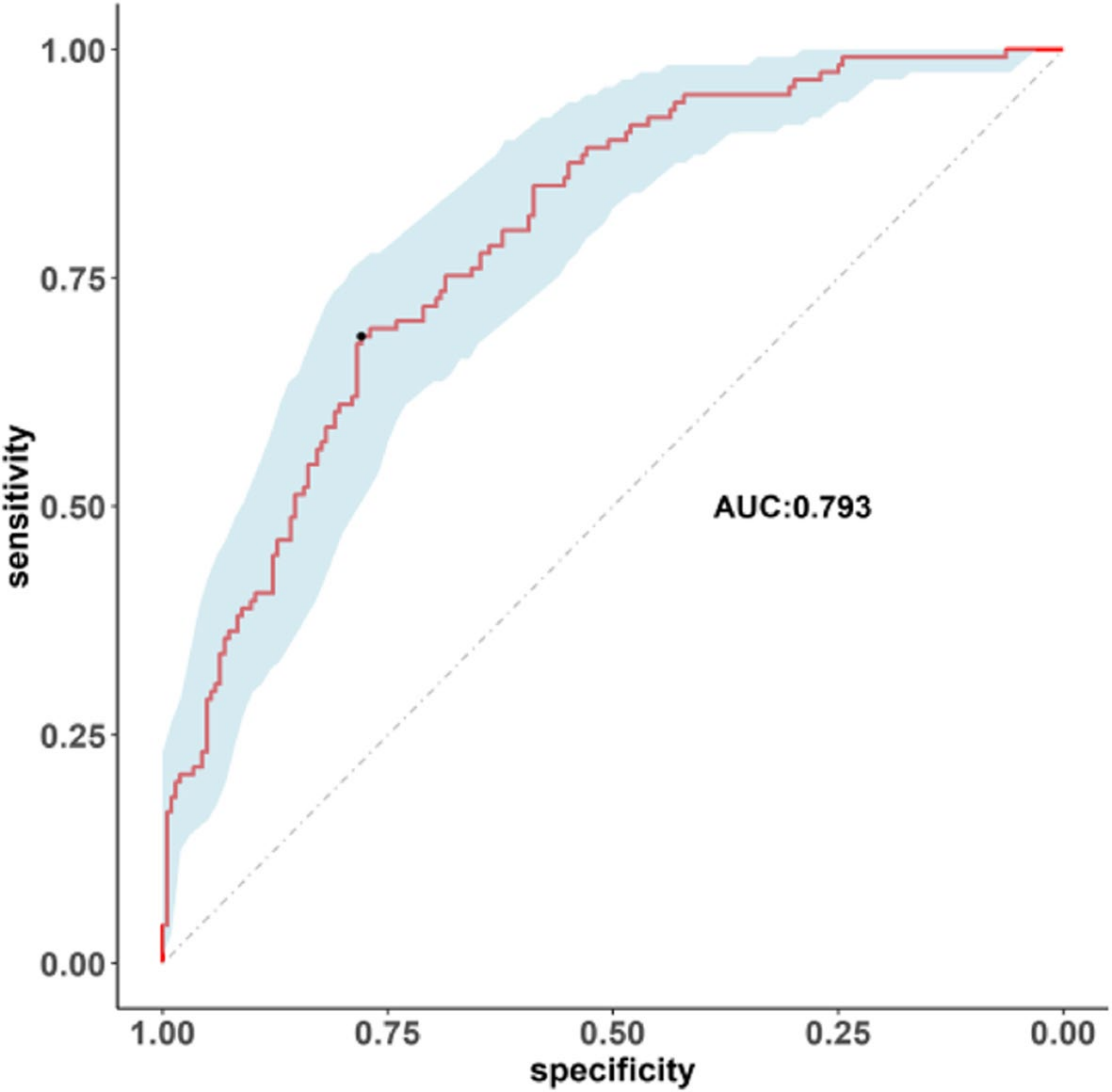
500 times of the Bootstrap ROC curve

The predicted probability of planned transfer to the ICU after hip arthroplasty in elderly individuals was expressed by P-m. According to the prediction probability P-m and the actual postoperative planned transfer to the ICU in the model set, the ROC curve of the P-m value was drawn, and the AUC was used to evaluate the discrimination of the prediction model. AUC is usually used to quantify a logical model [6]. The closer the AUC is to the value of 1, the better the discrimination of the model, and in clinical practice, when 0.7 < AUC < 0.9, the model has better discrimination [7]. The ROC curve of this model had an AUC of 0.793, the diagnostic threshold was 0.396, the sensitivity was 0.779, the specificity was 0.686, and the 95% CI (0.718–0.874) indicated that this model had good discrimination, as shown in Fig. 3. After 500 bootstrap internal validations, the ROC curve showed an AUC of 0.793 (95% CI (0.7447–0.8422)), which indicated that the model had good discrimination (Fig. 4).

#### Calibration

**Fig. 5:**
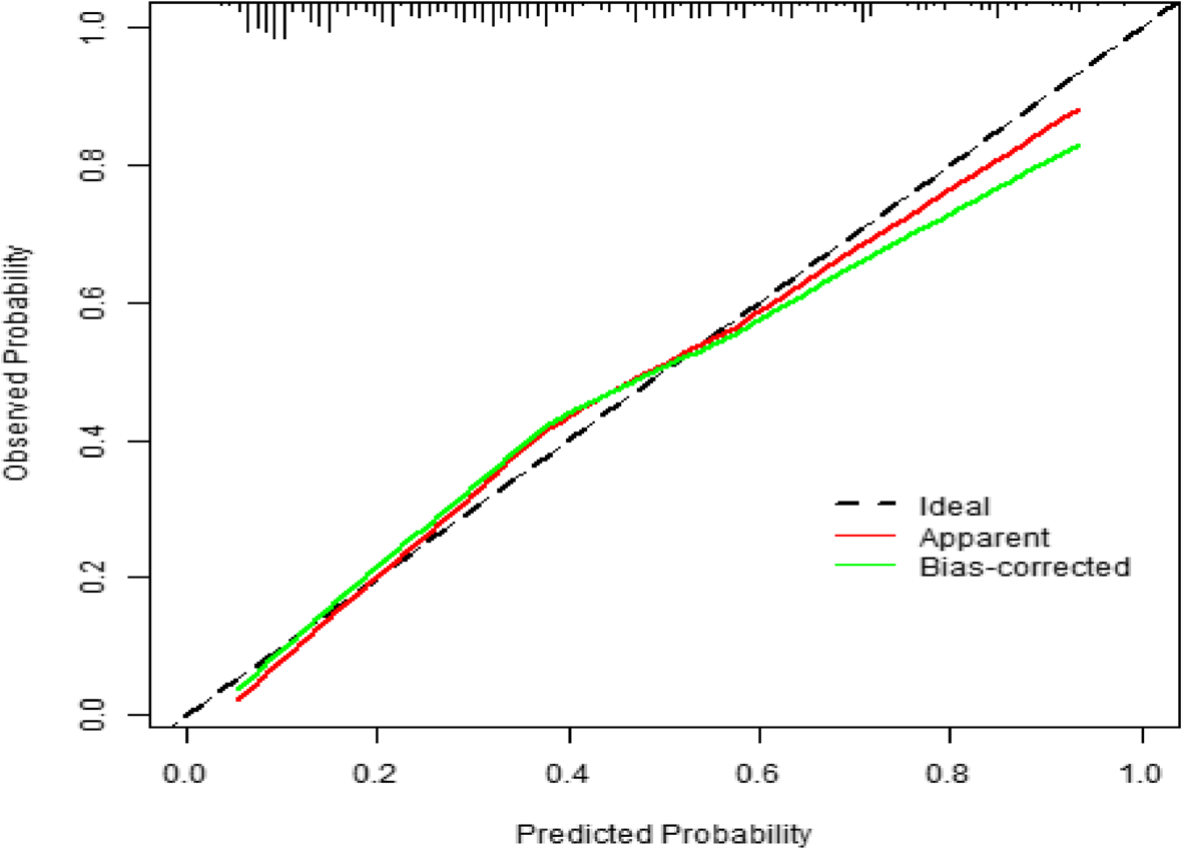
The calibration curve of the prediction model

Accuracy reflects the degree to which the model correctly estimates absolute risk, that is, whether the predicted value of the model is consistent with the actual value [8]. In this study, the calibration curve was established using the R language software package, and the Hosmer‒Lemeshow test was used for the internal test of calibration (Fig. 5). The x-axis represents the predicted probability of postoperative planned ICU transfer, and the y-axis represents the actual probability of postoperative planned ICU transfer. The ideal (diagonal) slope is 1, representing the ideal curve; apparent represents the uncalibrated prediction curve, and bias-corrected represents the calibrated prediction curve. The two curves fluctuate on the diagonal and do not deviate significantly from the ideal curve. The results of the Hosmer‒Lemeshow test showed that chi-square = 5.214611 (*P* = 0.8152131), and *P* > 0.1 indicated that the predicted value was in good agreement with the actual value, indicating that the prediction accuracy of this model was high.

#### Clinical usefulness

**Fig. 6:**
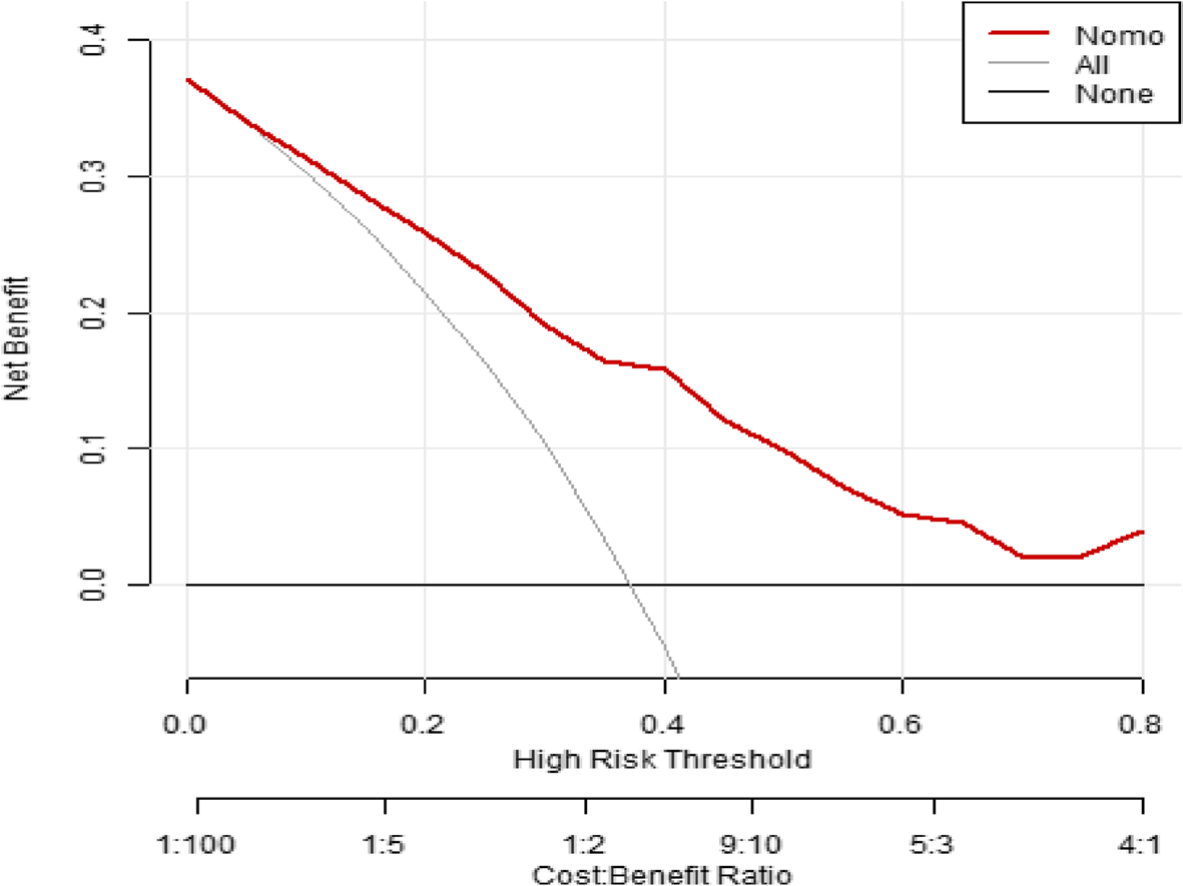
The DCA of the nomogram

Clinical practicability refers to the clinical net benefit of using the prediction model under a certain threshold probability, namely, DCA [9]. DCA obtains the net benefit value of using the model at the threshold by determining the relationship between the selected prediction probability threshold and the relative value of false-positive and false-negative results [10, 11]. The net benefit is calculated by all possible risk thresholds between the two extremes, namely, zero and maximum risk estimates, representing all negative events and all positive events, respectively [12]. If DCA is higher than two extreme lines, it indicates that patients can benefit and have better clinical practicability [13]. The black horizontal line in the DCA curve of this prediction model indicates that when all patients after hip arthroplasty are not transferred to the ICU, the clinical net benefit is zero; the grey line indicates that when all patients with hip arthroplasty are transferred to the ICU after the operation, the clinical net benefit has a negative slope; the red curve is based on the curve related to the prediction model in this study. When the prediction probability P-m is between 12 and 80%, the red curve is above the two extreme lines, indicating that the patients can benefit from the prediction, as shown in Fig. 6.

#### Model rationality re-evaluation

**Fig. 7:**
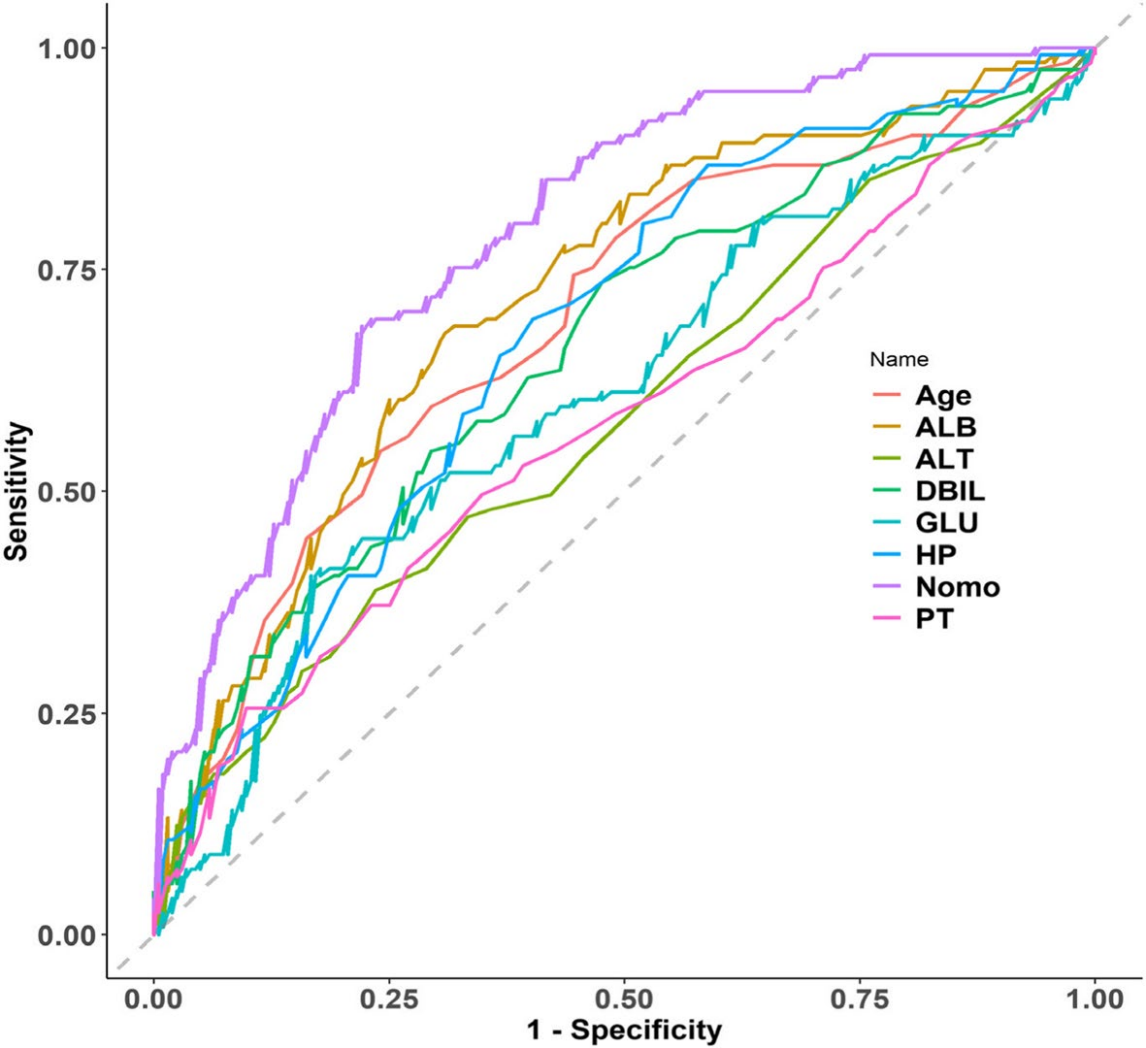
The ROC curve of nomogram and model internal factors. GLU Blood glucose, DBIL Direct bilirubin, ALT Glutamic-pyruvic transaminase, ALB Albumin, TP Total protein, PT Prothrombin time, HP Hemoglobin

**Fig. 8:**
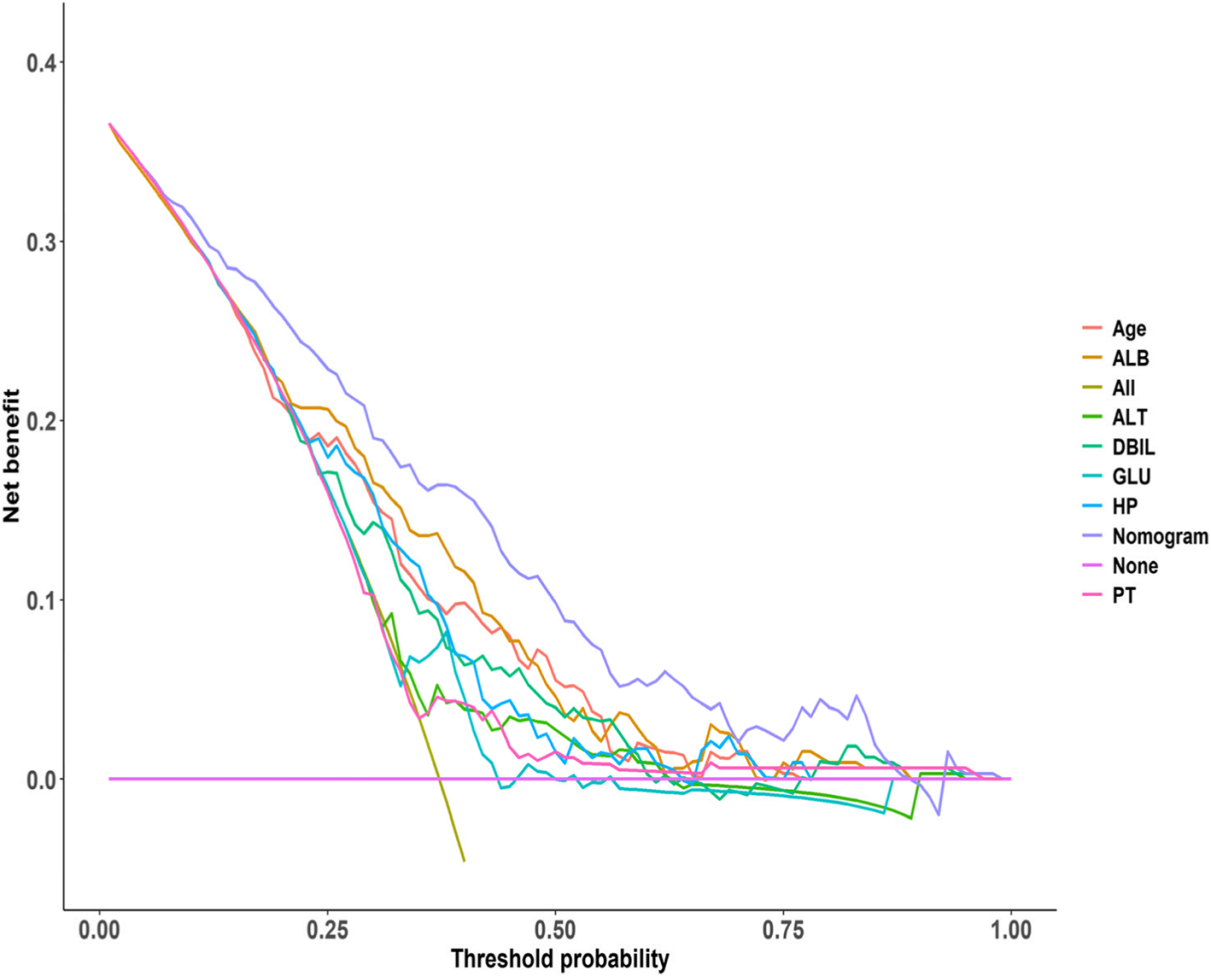
DCA curve of nomogram and model internal factors. GLU Blood glucose, DBIL Direct bilirubin, ALT Glutamic-pyruvic transaminase, ALB Albumin, TP Total protein, PT Prothrombin time, HP Hemoglobin

We compared the constructed nomogram prediction model with the seven factors in the model in terms of discrimination and clinical applicability. ROC curves and DCA curves were drawn to verify the rationality of the model (Figs. 7 and 8). The area under the ROC curve of the nomogram in Fig. 7 is larger than that of the other seven factors, indicating that this prediction model has the best discrimination among all models. The DCA curve of the nomogram in Fig. 8 is located at the outermost side of the internal factor curve, indicating that the clinical practicability of this prediction model is the best and worthy of clinical application. We evaluated the fit of the logistic regression model using pseudo-R squared, The Hosmer and Lemeshow R^2^ was 0.208, the Cox and Snell R^2^ was 0.24 and the Nagelkerke R^2^ was 0.327, respectively. This indicates a good fit of this model.

## Discussion

Hip arthroplasty is the most effective surgical intervention for the treatment of hip diseases and improvement of functional limitations. More than 1 million hip replacements are performed worldwide each year. As people’s life expectancy increases and health care advances, an increasing number of older patients undergo total hip arthroplasty (THA) [14], which increases the risk of postoperative complications. The functions of various systems of elderly patients gradually declined. Various basic diseases, such as those of the respiratory system, cardiovascular system and nervous system, often occur before surgery. These preoperative basic diseases can lead to or affect the occurrence of postoperative complications [15]. Studies have shown that the top three fatal complications after total hip arthroplasty are cardiovascular and cerebrovascular diseases and pulmonary embolism. Despite advances in surgical techniques and preoperative evaluation techniques, some patients undergoing hip arthroplasty surgery still need to be transferred to the ICU. The ICU is a new form of medical management with the emergence of new medical equipment, medical management systems and medical nursing professional development. It can provide continuous monitoring and comprehensive treatment support for critically ill patients in various departments of the hospital and provide material, human and technical support to the greatest possible extent [16]. Related studies have pointed out that compared with the conventional postoperative return to the ward, immediate transfer to the intensive care unit (ICU) after surgery, compared with timely rescue treatment when the patient’s condition changes, helps to reduce the mortality rate [17]. ICU admission is usually considered an important measure to prevent or treat life-threatening complications [18]. According to the relevantstudies, there is no systematic evaluation of the criteria for planned transfer to the ICU after hip arthroplasty in elderly patients. In this study, age, blood glucose, direct bilirubin, alanine aminotransferase, serum albumin, prothrombin time, and haemoglobin were independent factors affecting planned transfer to the ICU after hip arthroplasty in elderly individuals.

Age is usually included in the patient assessment as a risk factor in clinical work. Elderly patients are more likely to undergo hip arthroplasty. Age has been shown to be an important factor in major complications after total hip arthroplasty (THA) [19–23]. Age was found to be the strongest predictor of major postoperative complications [19]. Some studies also found that increased age was an independent risk factor for ICU admission [24]. In the multivariate analysis of this study, the OR value for age was 1.06 (95% CI (1.031–1.098)), which was consistent with the conclusions of previous studies. Age is an independent risk factor for planned transfer to the ICU after hip arthroplasty in elderly patients.

Blood glucose is an independent risk factor for planned ICU transfer after hip arthroplasty in elderly individuals. The OR value of blood glucose in this study was 1.07 (95% CI (0.982–1.167)). ther studies found that the OR value of blood glucose was 1.0057 (95% CI (1.0025, 1.0088)) in the risk factors for unplanned ICU transfer after joint replacement. As an independent influencing factor, blood glucose should be given full attention, whether transfer to the ICU after the operation is planned or unplanned. Higher preoperative blood glucose increases the risk of orthopaedic postoperative complications and surgical site infections. Hyperglycaemia is associated with impaired leukocyte function, including decreased phagocytosis, impaired bacterial killing, and impaired chemotaxis, ultimately leading to infection and poor wound healing by affecting the host’s immune system [25]. A number of studies have confirmed that perioperative blood glucose levels should be used as an important indicator of perioperative management of patients. Better blood glucose control can reduce surgical site infections and improve postoperative outcomes [26, 27]. It is generally required that the blood glucose level of patients undergoing elective major orthopaedic surgery be controlled at 6.0–11.1 mmol/L [28, 29].

The other three independent influencing factors in this study were direct bilirubin, alanine aminotransferase, and prothrombin time. All three indicators directly or indirectly reflect liver function. In multivariate logistic analysis, direct bilirubin had an OR of 1.12 (95% CI (1.023–1.236)), alanine aminotransferase had an OR of 1.03 (95% CI (1.011–1.061)), and prothrombin time had an OR of 1.12 (95% CI (0.995–1.287)). The OR values of the three influencing factors were all > 1, indicating that the three indicators in this study were positively correlated with planned transfer to the ICU after hip arthroplasty in elderly individuals; that is, an increase in the three indicators would increase the risk of planned transfer to the ICU after hip arthroplasty in elderly individuals. Recent studies have shown that a slight increase in bilirubin levels can effectively prevent cardiovascular disease and type 2 diabetes. The mechanism may be that bilirubin has strong antioxidant and anti-inflammatory effects [30, 31]. Direct bilirubin in this study (OR > 1) is a risk factor, which is inconsistent with the latest findings on the role of direct bilirubin. Because this study is mainly based on elderly patients, it may be due to advanced age, many previous underlying diseases, and long-term oral medication. It may also be related to the statistics of variable screening used in data analysis. In some studies on risk factors for ICU admission after joint replacement, alanine aminotransferase was *P* < 0.01 in baseline analysis. In further logistic regression analysis, alanine aminotransferase was not included in the risk factors, which may be related to the sample size or the statistical analysis method used. Prothrombin time is mainly determined by the level of coagulation factors I, II, V, VII, and X synthesized by the liver, and its role in liver disease is particularly important. All three indicators reflect liver function. In clinical work, it is necessary to know the patient’s hepatitis B condition to determine whether preoperative liver function intervention is needed. For elderly hip arthroplasty patients, the combination of these three factors should be considered to determine whether they should be transferred to the ICU.

Another independent risk factor was preoperative serum albumin. In this study, preoperative serum albumin had an OR of 0.93 (95% CI (0.874–0.982)), which was basically consistent with the OR of serum albumin in previous studies (OR = 0.90, 95% CI (0.84, 0.97)). Preoperative serum albumin is an independent influencing factor for planned transfer to the ICU after hip arthroplasty in elderly patients. Patients with hypoalbuminemia are more likely to have postoperative complications, especially delayed wound healing, pleural effusion and pneumonia. The risk of postoperative complications in patients with preoperative hypoalbuminemia (serum albumin < 35 g/L) was 1.89 times higher than in those with normal preoperative albumin (preoperative serum albumin ≥ 35 g/L) [32]. The decrease in serum albumin level is also related to the increase in average hospitalization days and readmission rate within 30 days and the increase in morbidity and mortality [33]. In practical work, preoperative and postoperative serum albumin is an indicator that clinicians focus on and use to evaluate the nutritional status of patients. Preoperative albumin can seriously affect postoperative recovery and wound healing and lead to other complications.

Another important independent factor was preoperative haemoglobin. Some studies dentified 130 unplanned ICU admissions in a cohort of 22,343 patients undergoing elective joint replacement. In this study, the authors determined that smoking, bone cement arthroplasty, general anaesthesia, allogeneic blood transfusion, higher C-reactive protein, higher BMI and lower haemoglobin levels were important independent risk factors for accidental admission to the ICU. In this study, preoperative haemoglobin had an OR of 0.98 (95% CI (0.97–0.999)), while in a study of hip and knee replacement, AbdelSalam et al. [34]. reported a haemoglobin OR of 0.95 (95% CI (0.85–1.07)), which is similar to this study, fully proving the importance of preoperative haemoglobin in the perioperative period of joint replacement.

Our current study also has a few limitations. First, this was a retrospective study. The risk of postoperative planned transfer to the ICU was analysed by extracting laboratory test results from the patient’s medical records. Electrocardiogram, echocardiography and other related examinations of elderly patients were not included in the analysis. Second, due to the limited sample size, the data processing was not split into a training set and validation set; although we used 500 Bootstrap for internal validation. We still need further validation and replication studies using larger, diverse patient populations to confirm the external validity of our findings. Third, there may be some indicators in clinical work that affect the risk factors for postoperative planned transfer to the ICU that are not included here.

## Conclusions

In summary, through univariate and multivariate logistic regression analyses, age, blood glucose, direct bilirubin, alanine aminotransferase, serum albumin, prothrombin time, and haemoglobin were independent influencing factors for planned ICU transfer after hip arthroplasty in elderly patients. By evaluating the discrimination, calibration and clinical practicability of the model, it was proven that the prediction model has good clinical prediction ability and clinical practicability. The model is presented in the form of a linear graph, which provides an effective reference for the individual risk assessment of patients.

## Data Availability

The datasets used and analysed during the current study available from the corresponding author on reasonable request.

## Abbreviations

ICU: Intensive care unit
ROC: Receiver operating characteristic curve;
CCI: Comorbidity index
RED: Red blood cell
HP: Hemoglobin
HCT: Hematocrit
PLT: Platelet
CRP: C-reactive protein
PT: Prothrombin time
INR: International normalized ratio
TT: Thrombin time
APTT: Activated partial thromboplastin timepartial thromboplastin time
FIB: Fibrinogen
TP: Total protein
ALB: Albumin
AST: Glutamic-oxalacetic transaminase
ALT: Glutamic-pyruvic transaminase
TBIL: Total bilirubin
DBIL: Direct bilirubin
BUN: Usea nitrogen
SCR: Serum creatinine
GLU: Blood glucose
